# Supplementary material for: MetaRibo-Seq measures translation in microbiomes
Source: Nat Commun. 2020 Jun 29;11:3268. doi: 10.1038/s41467-020-17081-z (PMC7324362; doi:10.1038/s41467-020-17081-z)
Supplement: Supplementary file 10 — Supplementary Data 7 [file 41467_2020_17081_MOESM10_ESM.zip › File2/Confidence_High_Taxonomy/386434_out.krona.html]

Javascript must be enabled to view this page.

members
magnitude
magnitudeUnassigned
count
unassigned
taxon
rank

386434\_out

84

superkingdom
84
2

phylum
1239
84

84
186801
class

order
186802
84

family
186803
83

1
841
genus
83

SRS012849\_contig\_number\_31169


SRS143148\_contig\_number\_25562SRS146764\_contig\_number\_33685SRS147346\_contig\_number\_64888
3
1263105
species


SRS011084\_contig\_number\_1301SRS011405\_contig\_number\_11623SRS013951\_contig\_number\_contig-100\_255.219757SRS014235\_contig\_number\_40217SRS014412\_contig\_number\_256SRS014459\_contig\_number\_608SRS014923\_contig\_number\_50SRS015065\_contig\_number\_352SRS015264\_contig\_number\_9937SRS015431\_contig\_number\_372SRS015578\_contig\_number\_48SRS015663\_contig\_number\_contig-100\_412.200977SRS015890\_contig\_number\_189SRS016335\_contig\_number\_42431SRS016753\_contig\_number\_2854SRS016954\_contig\_number\_20084SRS017433\_contig\_number\_88SRS018623\_contig\_number\_11637SRS018984\_contig\_number\_249SRS019068\_contig\_number\_37076SRS019161\_contig\_number\_40636SRS019381\_contig\_number\_20732SRS019397\_contig\_number\_908SRS019445\_contig\_number\_8591SRS023346\_contig\_number\_2930SRS024331\_contig\_number\_contig-100\_1052.204662SRS024388\_contig\_number\_7536SRS024492\_contig\_number\_9263SRS042628\_contig\_number\_35257SRS045645\_contig\_number\_22598SRS047014\_contig\_number\_23527SRS048164\_contig\_number\_28395SRS048870\_contig\_number\_12410SRS049896\_contig\_number\_8647SRS049959\_contig\_number\_377SRS050998\_contig\_number\_contig-100\_161.91204SRS051031\_contig\_number\_34821SRS052027\_contig\_number\_17768SRS056519\_contig\_number\_4692SRS058070\_contig\_number\_6157SRS058770\_contig\_number\_2613SRS063040\_contig\_number\_38242SRS064276\_contig\_number\_6380SRS064645\_contig\_number\_contig-100\_1257.20384SRS065504\_contig\_number\_32977SRS075021\_contig\_number\_7945SRS077335\_contig\_number\_745SRS077454\_contig\_number\_18287SRS097889\_contig\_number\_41313SRS097920\_contig\_number\_34SRS098827\_contig\_number\_5448SRS098881\_contig\_number\_9655SRS100021\_contig\_number\_1448SRS104311\_contig\_number\_28131SRS104693\_contig\_number\_11606SRS104975\_contig\_number\_1290SRS105153\_contig\_number\_2438SRS1055022\_contig\_number\_14436SRS142503\_contig\_number\_20568SRS142712\_contig\_number\_contig-100\_1446.36741SRS143070\_contig\_number\_1374SRS143085\_contig\_number\_2073SRS143417\_contig\_number\_21507SRS143722\_contig\_number\_14816SRS143780\_contig\_number\_347SRS144135\_contig\_number\_8944SRS144362\_contig\_number\_44501SRS144506\_contig\_number\_28SRS144714\_contig\_number\_29248SRS146812\_contig\_number\_7730SRS147271\_contig\_number\_contig-100\_392.233520SRS147977\_contig\_number\_9628SRS148091\_contig\_number\_contig-100\_75.85807SRS148159\_contig\_number\_43254SRS148424\_contig\_number\_19075SRS893259\_contig\_number\_1786SRS893288\_contig\_number\_3276SRS893327\_contig\_number\_10433SRS893342\_contig\_number\_contig-100\_255.62363
species
79
360807

186806
1
family

genus
1
1730

species
1897001
1

SRS148784\_contig\_number\_contig-100\_4.156532
